# Letter to the editor regarding “Covid-19 transmission in fitness centers in Norway - a randomized trial”

**DOI:** 10.1186/s12889-022-14800-7

**Published:** 2022-12-27

**Authors:** Morten Valberg, Jon Michael Gran, Corina S. Rueegg, Marissa LeBlanc

**Affiliations:** 1 Oslo Center for Biostatistics and Epidemiology, Oslo University Hospital, Nydalen, P. O. Box 4950, 0424 Oslo, Norway; 2 Clinical Trials Unit, Oslo University Hospital, Nydalen, P. O. Box 4950, 0424 Oslo, Norway; 3 Oslo Center for Biostatistics and Epidemiology, Department of Biostatistics, University of Oslo, Blindern, P.O. Box 1122, 0317 Oslo, Norway

**Keywords:** Non-pharmaceutical interventions, Randomized trial, Covid-19

## Abstract

In a recently published paper in BMC Public Health we read about a randomized trial on Covid-19 transmission performed in five fitness centers in Oslo, Norway, during the spring of 2020. In our opinion, this study has major shortcomings in design and methodology, which have not been addressed by the authors.

## Background

In a recently published paper in BMC Public Health we read about a randomized trial on Covid-19 transmission performed in five fitness centers in Oslo, Norway, during the spring of 2020 [1]. In our opinion, this study has major shortcomings in design and methodology, which have not been addressed by the authors.

## Main text

The most obvious problem with the study is the low Covid-19 incidence in the trial, which reflects the low number of infections in Oslo at the time [2]. Regardless of the reasons for the low level of infection, there is no real value of randomization when the number of infectious individuals is very low, and the trial duration is a short pre-specified interval. With a near absence of infection in the study population, it should also be clear that generalizability to other levels of infection is not possible.

Another problem with the study is that it largely disregards that Covid-19 is an infectious disease. Infection, by definition, happens within groups of individuals and populations. In this study, this could mean within fitness centers, or groups of individuals training together at the centers or even outside the centers. These dependencies should have been taken into account at both the design and analysis stage. Simulation studies have been suggested for informing proper design and sample size calculations in these settings [3].

Other problems particular to trials for non-pharmaceutical interventions (NPIs) for infectious diseases due to non-blinding and interference, e.g. that the treatment of one subject can affect the outcome of another [3, 4], are also ignored and not discussed. Furthermore, the authors do not argue for the relevance of the intention to treat (ITT) effect under the present drop-out and non-compliance rates. This is a non-inferiority trial, for which it is known that the ITT principle can be problematic [5].

Even if the trial had not been underpowered and conducted without any of the other problems above, it is unclear how the results of a trial like this can be generalized to fitness centers in a non-experimental setting, let alone to other settings with, for example, different disease prevalence, virus strain, level of immunity, season and other simultaneous NPIs in the wider population. How such trials should guide future policy is therefore not obvious.

## Conclusions

To perform appropriate studies of the effects of NPIs for infectious diseases, that can guide future policy making, the challenges in both study design and causal inference from such studies cannot be ignored. As it has been argued by others, naïvely designed trials can be worse than uninformative, they can be misinformative [6]. The authors conclude that their study "show that it is feasible to apply rigorous randomized testing of public health measures during an ongoing disease outbreak", but we come to another conclusion. What we should learn from this study is that rigorous randomized testing of public health measures during an ongoing infectious disease outbreak is not trivial and should not be approached as a trivial task.

## Data Availability

Not applicable.

## Abbreviations

ITT: Intention to treat
NPI: Non-pharmaceutical intervention
